# Laboratory Exposures to Staphylococcal Enterotoxin B

**DOI:** 10.3201/eid1009.040250

**Published:** 2004-09

**Authors:** Janice M. Rusnak, Mark Kortepeter, Robert Ulrich, Mark Poli, Ellen Boudreau

**Affiliations:** * United States Army Medical Research Institute of Infectious Diseases, Fort Detrick, Maryland, USA

**Keywords:** Staphylococcal enterotoxin B, conjunctivitis, agents of bioterrorism, laboratory exposures, occupational exposures, toxins, ocular exposures, gastrointestinal symptoms, cutaneous symptoms, synopsis

## Abstract

Staphylococcal enterotoxins are 23- to 29-kDa polypeptides in the bacterial superantigen protein family. Clinical symptoms from intoxication with staphylococcal enterotoxins vary by exposure route. Ingestion results in gastrointestinal symptoms, and inhalation results in fever as well as pulmonary and gastrointestinal symptoms. Review of occupational exposures at the U.S. Army Medical Research Institute of Infectious Diseases from 1989 to 2002 showed that three laboratory workers had symptoms after ocular exposure to staphylococcal enterotoxin B (SEB). Conjunctivitis with localized cutaneous swelling occurred in three persons within 1 to 6 hours after exposure to SEB; two of these persons also had gastrointestinal symptoms, which suggests that such symptoms occurred as a result of exposure by an indirect cutaneous or ocular route. Ocular exposures from SEB resulting in conjunctivitis and localized swelling have not previously been reported. Symptoms from these patients and review of clinical symptoms of 16 laboratory-acquired inhalational SEB intoxications may help healthcare workers evaluate and identify SEB exposures in laboratory personnel at risk.

## Abstract 2

First report of symptoms after ocular exposure to staphylococcal enteroxin B in the laboratory is detailed.

Staphylococcal enterotoxins are 23- to 29-kDa polypeptides in the bacterial superantigen protein family that act by cross-linking HLA-DR or DQ molecules and T-cell receptors. This cross-linking results in potentially pathologic levels of proinflammatory cytokines, such as tumor necrosis factor α, interleukin 2, and interferon-γ (*1**,**2*). Therefore, symptoms of mild exposure are anticipated to resemble T-cell–mediated recall responses, similar to a Mantoux skin test.

Staphylococcal enterotoxin B (SEB) is one of at least 15 antigenically distinct enterotoxin proteins (*3**,**4*). Clinical symptoms depend on the route of exposure. After inhalation of SEB, clinical features include fever, respiratory complaints (cough, dyspnea, and retrosternal discomfort or chest pain), and gastrointestinal symptoms; severe intoxication results in pulmonary edema, adult respiratory distress syndrome, shock, and death (*5**,**6*). Ingesting SEB may cause food poisoning within 1 to 6 hours of exposure, manifested as acute salivation, nausea, and vomiting, followed by abdominal cramps and diarrhea (*7**,**8*). As ingesting SEB does not typically result in pulmonary symptoms, gastrointestinal symptoms observed from inhalational intoxication are postulated to result from secondary oral ingestion of SEB concomitant with the inhalational exposure.

One laboratory incident that resulted in nine cases of inhalational intoxication to SEB and several other outbreaks of food poisoning from ingesting staphylococcal enterotoxins have been reported in the literature (*5*). Symptoms occurring after ocular exposure and localized cutaneous swelling or conjunctivitis from staphylococcal enterotoxins have not been reported. We report three cases of purulent conjunctivitis with localized facial swelling that occurred after ocular exposure to SEB in the laboratory. Two of the three patients also complained of gastrointestinal symptoms. The symptoms in these three mucocutaneous-acquired cases, and summary of symptoms from 16 laboratory-acquired inhalational intoxications with SEB, may help define the clinical spectrum that might be expected after SEB exposures. The full spectrum of clinical signs and symptoms of intoxication with SEB is important to healthcare workers evaluating persons with potential exposures to these agents, including in the context of bioterrorism. This discussion is also relevant to military practitioners, since SEB has been previously developed as an incapacitating biowarfare agent.

## Methods

During a review of occupational exposures evaluated in the Special Immunizations Clinic at the U.S. Army Medical Research Institute of Infectious Diseases from 1989 to 2002, clinical evaluations of three laboratory workers with symptoms of conjunctivitis and localized swelling after exposure to SEB were identified. Patient records and occupational exposure summaries were reviewed. Additionally, clinical histories of 16 persons with symptoms after inhalational intoxication with SEB, obtained from both that research facility's medical records and occupational exposure reports, were reviewed to summarize the spectrum of symptoms resulting from inhalational exposure to SEB.

## Results

### Patient 1

While injecting SEB into the endotracheal tube of a rabbit, a 22-year-old male laboratory worker sprayed approximately 150 µg of SEB onto his right glove. Sometime later, he recalled scratching his nose and the area adjacent to his right eye. Three hours after the incident, he noted irritation, pruritus, and a yellow discharge from his right eye. Nine to 12 hours after the incident, he had onset of gastrointestinal symptoms (nausea, abdominal cramps, and loose stools [approximately eight nonbloody loose stools over the next 8 hours]), nasal congestion, postnasal drip, and a self-reported fever. The following morning (approximately 18 h after the incident), he awoke with profound swelling of the right lower eyelid and passed three more loose stools. He did not have headache, chills, vomiting, cough, dyspnea, chest discomfort, or myalgias.

Physical examination was remarkable for diffuse hyperemia of the eye, mildly edematous conjunctiva inferiorly, and edema of the lower lid. The patient was given loperamide for control of diarrhea and sulfacetamide ophthalmic ointment to the right eye four times daily. Gastrointestinal symptoms resolved within 2 days, and the ocular symptoms, nasal congestion, postnasal drip, and febrile symptoms resolved within 4 days. The laboratory worker discontinued loperamide at day 2 and sulfacetamide ointment at day 4.

The amount of SEB in the spray was estimated to be <150 µg. However, the amount of SEB exposure to the right eye was even less, since only a portion of the solution was sprayed onto his hand and rubbed into his eye. The worker was wearing a Tyvek suit (DuPont, Wilmington, DE) and gloves at the time of the exposure but no goggles or respirator. As a consequence, safety measures were modified to recommend only Leur-Lock syringes (Baxter International Inc., Deerfield, IL) and to require eye protection and surgical masks while working with the toxin.

### Patient 2

During the reconstitution of SEB within a biosafety cabinet, a 20-year-old laboratory worker injected the contents of a syringe containing 500 µg of SEB into a sealed vial and was exposed when the solution in the vial came under pressure. Approximately 100 µL of SEB in solution foamed from the sealed vial. A small portion of the solution came into contact with the fourth finger on her left hand. She was not wearing gloves. She immediately washed the site with soap and water for 15 seconds and rinsed the soap from her hands. Before she dried her hands, she unconsciously rubbed her left eye with her left hand.

Within 6 to 9 hours of exposure, she had onset of a thick mucous discharge from her left eye, a swollen eye lid, nausea, and loose stools. She had no fever, headache, cough, dyspnea, chest discomfort, vomiting, or myalgias. She was seen at a local emergency room that evening, and was given gentamicin eye drops with a presumed diagnosis of "pink eye" and phenergan for nausea. She was told to remove her contact lenses. The following morning, she was seen in the Special Immunizations Clinic for evaluation for a potential occupational exposure, after reporting to her supervisor that her symptoms might be related to contact with SEB the previous day. Physical examination showed swelling of the left eyelid and discharge from the eye. The discharge was initially described as "long threads" and was subsequently noted to have a yellow color and tear-like appearance. Her symptoms of nausea and diarrhea continued; symptoms exacerbated with food intake. The gastrointestinal symptoms resolved in 3 days, and the ocular symptoms in 5 days.

The estimated syringe loss was 500 µg, but the amount of exposure was estimated to be <50 µg, since only a small portion of the solution was in contact with her hand. Because the toxin is extremely water soluble, the immediate washing of the hands should have effectively removed a large amount of the toxin.

### Patient 3

One hour after cleaning a dime-size amount of liquid, likely to have been SEB, found outside a biosafety cabinet, a 23-year-old laboratory technician noted bilateral eye irritation, conjunctival erythema, and an excessive yellow ocular discharge that continued throughout the day. She awoke the next morning (day 2) with facial swelling, persistent ocular symptoms, and a subconjunctival hemorrhage (possibly resulting from SEB transfer from hand to eye). She medicated herself with diphenhydramine and brompheniramine and noted improvement in her symptoms the following day (day 3).

On the morning of day 3, she visited the Special Immunizations Clinic for evaluation. At that time, the facial swelling had resolved, and the ocular symptoms had nearly resolved. She had no fever, headache, cough, dyspnea, chest discomfort, nausea, vomiting, diarrhea, or myalgias. Physical examination was remarkable for bilateral conjunctival injection and a 5-mm x 2-mm subconjunctival hemorrhage at the inferolateral margin of the right iris. Complete blood count and erythrocyte sedimentation rate were within normal limits. She was seen by her private ophthalmologist later that day, who recommended no specific treatment. Her symptoms resolved on day 4.

Subsequently, this patient had mild ocular erythema and irritation (no facial swelling or conjunctivitis) while in the laboratory where SEB studies were ongoing. These symptoms resolved immediately after she left the room, which suggests hypersensitivity. She was advised to avoid entering the laboratory when SEB was being used or consider prophylactic use of antihistamines to control symptoms.

### Inhalational Cases

Three historical events that occurred during the now disbanded U.S. offensive biologic warfare program resulted in inhalational exposures to SEB and subsequent intoxication. The spectrum of symptoms occurring in the three events is summarized in the Table.

**Table:** Signs and symptoms of inhalational intoxication to staphylococcal enterotoxin B

Signs and symptoms	Event 1^a^ (1963) N = 2	Event 2^b^ (1963) N = 4	Event 3 (1964) N = 10	Total (%)
Generalized				
Fever	2	4	9	15/16 (93.7)
Chills	2	2	9	13/16 (81.3)
Headache	2	2	9	13/16 (81.3)
Myalgia	2	1	8	11/16 (68.7)
Fatigue	ND^c^	2	8	10/14 (71.4)
Malaise	ND	2	7	9/14 (64.3)
Lower respiratory				
Cough	2	3	10	15/16 (93.7)
Dyspnea	2	2	4	8/16 (50.0)
Retrosternal or chest pain	ND	3	5	8/14 (57.1)
Wheezing	1	0	1	2/16 (12.5)
Gastrointestinal				
Nausea	2	4	6	12/16 (75.0)
Vomiting	2	3	4	9/16 (56.3)
Anorexia	2	2	5	9/16 (56.3)
Abdominal cramps	ND	1	2	3/14 (21.4)
Diarrhea^d^	2	0	0	2/16 (12.5)
Gas	ND	0	1	1/14 (7.1)
Hepatitis	0	0	1	1/14 (7.1)
Upper respiratory				
Pharyngeal injection	ND	2	3	5/14 (35.7)
Rhinorrhea, postnasal drip, or sinus congestion	ND	2	2	4/14 (28.6)
Sore throat	ND	1	2	3/14 (21.4)
Otitis	ND	1	1	2/14 (14.3)
Hoarseness	ND	0	1	1/14 (7.1)
Other				
Conjunctival injection	ND	2	2	4/14 (28.6)
Burning eyes	ND	0	1	1/14 (7.1)
Flushed face	ND	1	0	1/14 (7.1)

^a^Only occupational summary reports reviewed (medical records not available). ^b^No records available on the one nonhospitalized symptomatic person. ^c^ND, no data. ^d^Loose stools noted in one person in the second and the third events.

In early 1963, two persons were exposed to SEB as a result of a ruptured hose that contained a crude filtrate of SEB under moderate pressure. Acute asthmatic bronchitis developed in one of these persons within a few hours of exposure. Fever, headache, myalgias, nonproductive cough, dyspnea, nausea, vomiting, and diarrhea developed in both persons, with maximal symptoms at 12 hours and resolution of symptoms by day 3.

In June 1963, five of seven persons became ill within 24 hours of exposure to a highly purified SEB aerosol after a dose-titration experiment in monkeys using a Henderson apparatus for administration of the aerosol; four of the persons required hospitalization (*9*). The source was suspected to be residual SEB from fur on the monkeys' heads, which had not been wiped after the exposure to SEB. Fever, cough, sternal tightness, anorexia, nausea, and vomiting were prominent features of intoxication; these signs and symptoms started within a few hours to as late as 24 hours after exposure.

The third event occurred in August 1964, when a leak in tubes carrying aerosolized SEB to test monkeys resulted in exposure of 15 persons. Ten persons became symptomatic, and 9 of them were hospitalized (*5*).

Onset of symptoms from inhalational intoxication was within 1–1/2 hours to 24 hours of exposure (most within 12 hours of exposure), and the duration of symptoms was generally 3–5 days. In addition to the previously reported and commonly observed symptoms of fever, headache, myalgias, pulmonary symptoms, and gastrointestinal symptoms, fatigue and malaise were observed in most persons (Table). While diarrhea was reported in a few cases, it was not a prominent finding with SEB intoxication by inhalation. Conjunctival injection, previously reported in the literature, was noted only in four persons.

Newly reported symptoms include upper respiratory symptoms (e.g., sore throat, profuse postnasal drip, sinus congestion, rhinorrhea, coryza, and hoarseness). Pharyngeal injection (five persons) and injected tympanic membranes (two persons) were observed; neither had been previously reported in the literature.

## Discussion

Outside the context of food poisoning, few physicians would be expected to have experience evaluating persons with SEB intoxication. However, an increase in laboratory exposures and intoxications with staphylococcal enterotoxins can be expected as additional institutions begin work with them as a result of increased funding for biodefense research. Symptoms of conjunctivitis with periocular or facial swelling, acquired by ocular or cutaneous exposure routes, have not been previously reported in the literature. A historical occupational health Department of Defense report suggests that conjunctivitis and upper respiratory tract symptoms resulting from exposures to staphylococcal enterotoxins were recognized during the time of the U.S. offensive biological warfare program from 1945 to 1969 (*10*). Therefore, documenting the full clinical spectrum of intoxications with staphylococcal enterotoxins is necessary to educate healthcare workers and safety officers to enable them to identify workers at risk and prevent exposures to staphylococcal enterotoxins.

Clinical symptoms from SEB may vary and are dependent on the dosage and route of exposure (*5*). While inhaling SEB may result in fever, pulmonary, and gastrointestinal symptoms; ingestion of staphylococcal enterotoxins generally results mainly with gastrointestinal symptoms. The gastrointestinal symptoms noted in two persons with ocular or percutaneous exposures (or both) suggest that gastrointestinal symptoms from SEB may occur by a nonoral route, although transmission of SEB to the gastrointestinal tract via the lacrimal duct cannot be entirely excluded. Also, recurring symptoms of ocular irritation and erythema when in the presence of SEB, and immediate resolution of symptoms when no longer in an SEB area, suggests a possible hypersensitivity to SEB.

The pathophysiology of symptoms from staphylococcal enterotoxins, however, is not fully understood. Staphylococcal enterotoxins are superantigens that act by cross-linking HLA-DR or DQ molecules and T-cell receptors, resulting in high levels of inflammatory cytokines such as interleukin 2, interferon-γ, and tumor necrosis factor (*1*). Staphylococcal enterotoxins resist inactivation by gastrointestinal proteases; oral dosages as low as 5–20 µg induce emesis in nonhuman primates (*4**,**8*). However, nongastrointestinal routes such as intravenous administration of SEB (higher dosages of 20 to 500 µg) also induced emesis in nonhuman primates.

High levels of cytokines alone may cause symptoms similar to SEB intoxication. Cancer patients, given high doses intravenously of the cytokine interleukin-2, have symptoms of fever, malaise, nausea, vomiting, and diarrhea, similar to SE food poisoning (*7*). Also, intravenous OKT3, a monoclonal antibody used as an immunosuppressant in transplant patients (it binds to T lymphocytes, resulting in early activation of T cells, cytokine release, and subsequent blocking of T-cell functions), has a side effect profile similar to that of SEB—high fever, gastrointestinal symptoms, arthralgias, and pulmonary symptoms (*11*).

The mechanism of emesis also has been postulated to be related to the stimulation of mast cells and the subsequent release of cysteinyl leukotrienes and histamine (*12*). L4 171883, a selective inhibitor of LTD4/LTEF receptors, completely eliminated the emetic response and immediate type skin reactions (skin reactions associated with degranulation of cutaneous mast cells) to SEB. Inhibition of prostaglandins by indomethicin or pretreatment with a dual lipoxygenase and cyclo-oxygenase inhibitor (BW 755C) did not prevent emesis or immediate type skin reactions. After degranulation of mast cells, impulses are sent through the vagus and sympathetic nerves to the medullary center, which results in emesis. Severing of the vagus and sympathetic nerves inhibits the emesis response (*13*).

The mechanism of diarrhea is even less well understood, although it is not by means of activation of adenylate cyclase (*5*). Histopathologic findings with staphylococcal food poisoning are minimal; they mainly show polymorphonuclear cell infiltrates in the epithelium and lamina propria of the stomach and proximal small intestine (*7**,**8*).

SEB intoxication is diagnosed by clinical symptoms and a history of potential exposure to SEB. Definitive diagnosis of inhalational exposure can be made by nasal swabs and induced respiratory secretions for toxin assays, blood and urine for immunoassay, and acute- and convalescent-phase serum, but these tests are not readily available and not reliable for low-dose exposures. While inhalational intoxication with SEB is generally associated with leukocytosis and a mildly elevated erythrocyte sedimentation rate, these findings are nonspecific. Chest x-ray findings of increased interstitial markings, atelectesis, overt pulmonary edema, or acute respiratory distress syndrome are also nonspecific and only present in inhalational intoxications of SEB (*5*). Potential changes in serum antibody titers, although relevant, have not been examined.

Toxic and lethal doses of SEB vary greatly between animal species, mostly because of differences in receptor-binding affinities, and also vary depending on the route of exposure (*14*). In humans, the estimated 50% lethal dose (LD_50_) is 0.02 µg/kg and 50% effective dose (ED_50_) is 0.0004 µg/kg by aerosolized exposure (*14*). No data exist on the LD_50_ and ED_50_ in humans by other routes of exposure. The ED_50_ is estimated to be 0.03–0.26 µg/kg in monkeys and 12–40 µg in chimpanzees, by intraperitoneal or intravenous challenge. The extrapolation of the estimated values of ED_50_ of nonhuman primates to humans would suggest that 2 µg versus 840 µg of SEB would be needed to cause symptoms in a 70-kg person through the ocular or cutaneous route. Occurrence of symptoms in two persons after exposure to dosages of SEB <50 µg provides support that the lower ED_50_ value in monkeys may also apply to humans.

During the offensive biologic warfare program, a contractor report addressing the efficacy of biosafety cabinets noted toxic reactions in persons performing SEB purification studies on open laboratory benches (*10*). The following symptoms were noted in six persons: conjunctivitis, nondescript chemical irritation of one eye, general skin reaction, severe facial skin reaction, dermatitis, and cold symptoms. Additionally, symptoms mainly of conjunctivitis and acute pharyngitis, but also including vomiting and diarrhea in two cases, were observed in 23 persons wearing surgical masks or face shields while working with SEB. Persons working with SEB within a biosafety cabinet had no symptoms.

As exposure to low dosages of SEB can produce symptoms, these recently reported symptoms have importance both to safety officers and healthcare workers evaluating laboratory workers at risk with potential exposures to staphylococcal enterotoxins. SEB intoxication can mimic an infectious process. The initial diagnosis of the first person who sought medical evaluation in the June 1963 incident was pneumococcal pneumonia; symptoms included acute onset of fever, chills, productive cough, chest pain, and dyspnea. The patient was started on penicillin, which was discontinued after his co-workers exhibited similar symptoms, a finding that supported the diagnosis of SEB intoxication. Even though medical providers had knowledge of SEB exposure in a subsequently hospitalized patient involved in the June 1963 incident, the initial symptoms of this patient still raised concern about a possible infectious cause. That patient was noted to have a flushed face, mild hyperemia of the pharynx, a prominent postnasal drip, a purulent-appearing otitis media and externa without symptoms of ear pain, pulmonary symptoms (productive cough and chest discomfort), and a leukocytosis of 19,500 cells/mm^3^. Their differential diagnosis included otitis externa and media, pneumonia, or SEB intoxication. Otic examination was within normal limits 24 hours later, which suggested SEB as the possible cause of the localized swelling. An infectious cause was also considered as the initial primary diagnosis in the initial two patients with conjunctivitis in this series as the cause of the conjunctivitis, gastrointestinal symptoms, or both, with both persons receiving topical ophthalmic antimicrobial agents for conjunctivitis. Healthcare workers evaluating persons who work with SEB need to be aware of the full spectrum of toxicity symptoms associated with SEB to avoid misdiagnosis resulting in unnecessary treatment, to identify breaches in laboratory technique, and to educate persons at risk of the importance of personal protective measures in preventing SEB exposure and intoxication. These cases emphasize that personal protective measures such as biosafety cabinets, gloves, and eye protection are paramount when working with SEB.
